# *Limosilactobacillus fermentum* KAU0021 Abrogates Mono- and Polymicrobial Biofilms Formed by *Candida albicans* and *Staphylococcus aureus*

**DOI:** 10.3390/pharmaceutics15041079

**Published:** 2023-03-27

**Authors:** Irfan A. Rather, Mohmmad Younus Wani, Majid Rasool Kamli, Jamal S. M. Sabir, Khalid Rehman Hakeem, Ahmad Firoz, Yong Ha Park, Yan Yan Hor

**Affiliations:** 1 Department of Biological Sciences, Faculty of Science, King Abdulaziz University, Jeddah 21589, Saudi Arabia; 2 Center of Excellence in Bionanoscience Research, King Abdulaziz University, Jeddah 21589, Saudi Arabia; 3 Department of Biotechnology, Yeungnam University, Gyeongsansi 38541, Republic of Korea; 4 Department of Chemistry, College of Science, University of Jeddah, Jeddah 21589, Saudi Arabia; 5 Probionic Corporation, Jeonbuk Institute for Food-Bioindustry, 111-18, Wonjangdong-gil, Deokjin-gu, Jeonju-si 54810, Republic of Korea

**Keywords:** *Staphylococcus aureus*, *Candida albicans*, *Lactobacillus*, polymicrobial biofilms, membrane disintegration

## Abstract

*Candida albicans* and *Staphylococcus aureus*, representing two different kingdoms, are the most frequently isolated pathogens from invasive infections. Their pathogenic attributes, combined with drug resistance, make them a major threat and a challenge to successful treatments, mainly when involved in polymicrobial biofilm-associated infections. In the present study, we investigated the antimicrobial potential of *Lactobacillus* metabolite extracts (LMEs) purified from cell-free supernatant of four *Lactobacillus* strains (KAU007, KAU0010, KAU0021, and Pro-65). Furthermore, LME obtained from the strain KAU0021 (LME^KAU0021^), being the most effective, was analyzed for its anti-biofilm property against mono- and polymicrobial biofilms formed by *C. albicans* and *S. aureus*. The impact of LME^KAU0021^ on membrane integrity in single and mixed culture conditions was also evaluated using propidium iodide. The MIC values recorded for LME^KAU0021^ was 406 µg/mL, 203 µg/mL, and 406 µg/mL against planktonic cells of *C. albicans* SC5314, *S. aureus* and polymicrobial culture, respectively. The LME^KAU0021^ at sub-MIC values potentially abrogates both biofilm formation as well as 24 h mature mono- and polymicrobial biofilms. These results were further validated using different microscopy and viability assays. For insight mechanism, LME^KAU0021^ displayed a strong impact on cell membrane integrity of both pathogens in single and mixed conditions. A hemolytic assay using horse blood cells at different concentrations of LME^KAU0021^ confirmed the safety of this extract. The results from this study correlate the antimicrobial and anti-biofilm properties of lactobacilli against bacterial and fungal pathogens in different conditions. Further in vitro and in vivo studies determining these effects will support the aim of discovering an alternative strategy for combating serious polymicrobial infections caused by *C. albicans* and *S. aureus*.

## 1. Introduction

Increasing incidences of polymicrobial infections caused by fungi and bacteria in various hospital settings have been widely reported. *Candida albicans* and *Staphylococcus aureus* are the most frequently isolated microbes from invasive coinfections globally [1,2]. The medical implications associated with coinfections are a matter of grave concern; factors such as a limited treatment regimen, mainly when multi-drug resistant (MDR) strains (methicillin-resistant *S. aureus*, MRSA) are involved in biofilm formation, are responsible for high rates of mortality in immunocompromised patients [1,3,4,5].

Biofilms are composed of one or more types of microbes that adhere to many surfaces, including biotic or abiotic surfaces, and act as a potential source for spreading infection. A biofilm is a three-dimensional structure with microbes embedded in an extracellular matrix (ECM); the sessile cells have reduced sensitivity towards antimicrobial agents, unlike their planktonic counterparts. This property is mainly responsible for the endurance of infections and contributes to treatment failure [6,7]. Notably, echinocandins have been registered as a first line of therapy for combating *Candida* species infections, especially biofilm-associated ones [8]. In addition, reports are available for drugs that can be used as efflux pump inhibitors to reduce biofilm formation in *S. aureus* [9]. Researchers have outlined potential therapeutic strategies for treating polymicrobial biofilm infections caused by *C. albicans* and *S. aureus* [9,10]. However, tackling monomicrobial or polymicrobial biofilm infections with the current treatment options remain challenging for the therapeutic world. Therefore, finding effective and safe treatment strategies that target mono- and polymicrobial biofilms becomes crucial.

The family of lactic acid bacteria (LAB) is a diverse cluster of thirteen different bacterial genera that falls within the phylum *Firmicutes* [11]. Besides the role of LAB in food preservation, their antifungal and antibacterial activity has been widely explored by researchers. Lactic acid bacteria are well accepted for their role in the health industry as probiotics and are known to produce antimicrobial compounds such as bacteriocins, biosurfactants, and bacteriocin-like peptides (BLIS) [12,13,14,15]. Lactobacilli are members of the lactic acid bacteria and are one of the number of probiotics that are accepted as biological therapeutics and host immune-modulating entities that are generally recognized as safe (GRAS). In the past, researchers established several antimicrobial modes of action of *Lactobacillus*, such as competing for nutrients and binding sites, production of inhibitory compounds and host immune-stimulation [16]. Furthermore, beside producing lactic acid, *Lactobacillus* can also produce acetic acid, formic acid and so on to reduce intestinal pH, which may also play an important role in antimicrobial mechanisms; secretion of some molecules, such as ethanol, fatty acid, hydrogen peroxide and bacteriocins makes them good candidates for the antimicrobial drug development [17]. On a similar context, *L. casei*, *L. acidophilus* and *L. reuteri* showed an antibacterial effect against *Pseudomonas aeruginosa* [18]. Furthermore, Chen and co-workers (2019) screened 57 *Lactobacillus* isolates. Of these, five (LUC0180, LUC0219, LYC0289, LYC0413, and LYC1031) isolates displayed strong inhibitory activity against carbapenem-resistant *Enterobacteriaceae* [16]. Thus, *Lactobacillus* species has been widely studied for its antimicrobial activity and few compounds isolated from them have been fully or partially characterized [19,20,21,22]. Despite this, each *Lactobacillus* strain is believed to produce its peculiar compounds due to its unique characteristics. Furthermore, the anti-biofilm property of *Lactobacillus* strains against oral pathogens [23] and vaginal microbiota [24] has been well documented in the literature. Therefore, the present study aims to expand the antimicrobial property of *Lactobacillus* strains against mono- and polymicrobial infections. In the present study, we evaluated the antifungal and antibacterial potential of *Lactobacillus* metabolite extracts (LMEs) purified from cell-free supernatant of four *Lactobacillus* strains (KAU007, KAU0010, KAU0021, and Pro-65). Furthermore, LME obtained from the strain KAU0021 (LME^KAU0021^), being the most effective one, was analyzed for its anti-biofilm properties against monomicrobial and polymicrobial biofilms formed by *C. albicans* and MRSA. In addition, the impact of LME^KAU0021^ on cell viability and membrane integrity was evaluated. Moreover, it is advantageous to correlate the antimicrobial properties of lactobacilli with anti-biofilm properties against mono- and polymicrobial biofilms to discover an alternative strategy for combating serious infections caused by *C. albicans* and MRSA under mono- and polymicrobial conditions.

## 2. Materials and Methods

### 2.1. Identification and Characterization of the Lactobacillus Strains

*Lactiplantibacillus plantarum* KAU007 was isolated from camel milk in Jeddah, Saudi Arabia [23]. *Lactiplantibacillus pentosus* KAU0010 and *Limosilactobacillus fermentum* KAU0021 were isolated from fermented ajawa dates of Madinah by following the same method as described elsewhere [24]. The isolation and molecular characterization were carried out following the same procedure described elsewhere [25]. *Lactobacillus sakei* Probio-65, a well-known commercial probiotic strain isolated from Kimchi, was provided by Dr. Lew Lee Ching, ProBionic, Republic of Korea. The 16S rRNA gene sequences of KAU0010 and KAU0021 were submitted to GenBank for accession numbers.

### 2.2. Pathogenic Strains and Growth Conditions

In this study, laboratory-stored isolates of *Candida albicans* SC5314 and *Staphylococcus aureus* ATCC29213 were used. Both strains were initially revived from glycerol stocks on sabouraud dextrose agar (SDA; Sigma-Aldrich, St. Louis, MO, USA) for *C. albicans* SC5314 and nutrient agar (NA; Sigma-Aldrich, USA) for *S. aureus* ATCC29213.

### 2.3. Preparation of Lactobacillus Metabolite Extract

All four strains such as *Lactiplantibacillus plantarum* KAU007, *Lactiplantibacillus pentosus* KAU0010, *Limosilactobacillus fermentum* KAU0021 and *Lactobacillus sakei* Probio-65, hereafter named as KAU007, KAU0010, KAU0021, and Pro-65, respectively, were propagated onto De Man, Rogosa and Sharpe (MRS; Sigma-Aldrich, USA) agar plates and incubated at 30 °C for 48–72 h. Thereafter, single colony grown on MRS agar plates was picked up, inoculated in MRS broth and incubated at 30 °C for 24 h. The broth was centrifuged, then the pellet was recovered and washed with PBS. The pellet was dissolved in PBS, and the cell concentration was adjusted to 0.5 McFarland standard (1.5 × 10^8^ CFU/mL) by using a turbidimeter; this was used as a standard inoculum for secondary metabolite preparation. For the production of secondary metabolites, a method described by Badwaik and coworkers [26] was adopted. Briefly the MRS broth was inoculated with *L. plantarum* KAU007 at 2% of the standard inoculum (*v*/*v*) concentration and kept at 30 °C for 48 h at 150 rpm. Afterwards, the fermented media was centrifuged (4400 rpm for 20 min at 4 °C) to secure the cell-free supernatant (CFS). The CFS was then filtered using 0.22 μm vacuum filtration system (Nalgene, Rochester, NY, USA) to remove any remaining cells. The secondary metabolites from CFS were finally extracted using solvents of different polarities (EA; Sigma-Aldrich, USA). Post-extraction, the organic layers, along with the interfacial layers, were collected and evaporated using a rotary vacuum evaporator (Buchi, Germany), and the LMEs were vacuum-dried. A working solution of LMEs (10 mg/mL) was prepared in 1% dimethyl sulfoxide (DMSO; Sigma-Aldrich, USA) and was used to evaluate antimicrobial activity and anti-biofilm activity.

### 2.4. Antimicrobial Activity of LMEs

The antimicrobial activity of LMEs was evaluated by determining the minimum inhibitory concentrations (MIC) against *C. albicans* SC5314 and *S. aureus* ATCC29213. The broth microdilution assay was employed for the determination of the MIC values of LMEs (LME^KAU0010^, LME^KAU0021^, LME^KAU007^, LME^Pro−65^) against planktonic cells of *C. albicans* SC5314 [26] and *S. aureus* ATCC29213 [27]. Briefly, inoculum was prepared by growing *C. albicans* and *S. aureus* in Sabouraud Dextrose Broth (SDB; Sigma-Aldrich, USA) and cation-adjusted Mueller–Hinton broth (MHB; Sigma-Aldrich, USA) respectively at 37 °C for 24 h. The yeast and bacterial cells were centrifuged and resuspended in respective media and the turbidity of the suspension was adjusted to 0.5 McFarland Standard (equivalent to 1.0–5.0 × 10^6^ CFU/mL, yeast; 1.0–2.0 × 10^8^ CFU/mL, bacteria) using a MicroScan Turbidity meter (Beckman Coulter, Brea, CA, USA). The final inoculum concentration was further adjusted to 1 × 10^3^–5 × 10^3^ CFU/mL and 5.0 × 10^5^ CFU/mL for yeast and bacteria, respectively. The tested concentration of all the LMEs ranged from 6496 to 6.34 µg/mL. The experiment was performed in 96-well microtiter plates containing 100 μL of 2-fold serial dilutions of LMEs in 100 μL respective growth medium for yeast and bacteria. The plates were then inoculated with 100 μL of the inoculum and incubated for 24 h at 37 °C. The lowest concentration of LMEs resulting in the inhibition of visible microbial growth was calculated as their MIC. Following MIC determination, the minimum bactericidal concentration (MBC) and minimum fungicidal concentration (MFC) of all the LMEs against *S. aureus* and *C. albicans* was determined. For this purpose, each well without growth in MIC plates, yeast and bacteria was sub-cultured onto SDA and MHA plates, respectively, followed by incubation at 37 °C for 24 h. The minimum—cidal concentrations were calculated as the lowest concentrations of LMEs that destroyed around 99.9% of microbial cells, compared to their respective negative controls [27]. The ethyl acetate extract metabolites showed the best results. Azithromycin (Sigma-Aldrich, USA) [28] and fluconazole (Sigma-Aldrich, USA) [29] were used as positive controls against bacterial and fungal isolates, respectively. In addition, 1% DMSO was used as a vehicle control in the experiment.

### 2.5. Antimicrobial Activity of LMEs against Mixed Microbial Population

After determining the susceptibility of LMEs against individual pathogens, MICs of the LMEs against planktonic cells of *S. aureus* and *C. albicans* under mixed conditions were evaluated by following the protocol published previously [30]. Briefly, 100 µL of two-fold dilution of each LME, 50 µL of *C. albicans* SC5314 (2 × 10^3^ cells/mL), and 50 µL of *S. aureus* inoculum (2 × 10^5^ CFU/mL) were added to the flat-bottomed 96-well microtiter plate and incubated at 37 °C for 48 h and MICs were calculated as described above.

Furthermore, after determining the MICs in mixed cultures, MBC/MFC values were also calculated for mixed cultures, as described above.

### 2.6. Assessment of Anti-Biofilm Activity of LMEs on Mono- and Polymicrobial Biofilms

Since LME^KAU0021^ displayed potent antimicrobial activity against yeast and bacteria, it was selected for further in-depth study. Herein, the anti-biofilm activity of LME^KAU0021^ was determined in two different growth phases—at the time of biofilm formation and on mature biofilms of *C. albicans* and *S. aureus*. The anti-biofilm potential against monomicrobial and polymicrobial biofilms of the test pathogens was evaluated as described previously [31] with some modifications. Briefly, the inoculum for yeast and bacteria was prepared at a concentration of 1 × 10^6^ CFU/mL in RPMI 1640-MOPS (Sigma-Aldrich, USA) and Brain Heart Infusion (BHI; Diagnostic Media Products, Johannesburg, South Africa), respectively. Monomicrobial biofilm was established by adding 100 µL of microbial cell suspension in 100 µL of the respective medium in designated flat-bottomed 96-well polystyrene microplate. At the same time, the polymicrobial biofilms were allowed to form by adding 50 µL of each bacterial and yeast suspension to 100 µL of Tryptic Soy Broth (TSB; Sigma-Aldrich, USA); TSB and BHI have been previously determined to be optimal media for supporting both *C. albicans*/*Staphylococcus aureus* (dual species) biofilm [32]. The plate was then incubated at 35 °C with mild shaking, 100 rpm for 90 min (adhesion phase). Next, the growth media was removed, sessile cells were washed gently with PBS, and 100 µL of the respective medium was added to their designated wells to allow biofilm formation by incubating the plate at 35 °C, 100 rpm for 24 h. The untreated cells were considered negative controls, and the medium alone was used as sterility control. Additionally, to determine the anti-biofilm property, 100 µL of respective medium supplemented with LME^KAU0021^ (6496 to 6.34 µg/mL) was added to the designated wells followed by the adhesion phase, and plates were incubated at biofilm-forming conditions.

A parallel experiment was designed to evaluate the anti-biofilm potency of test agents on mature biofilms. In this regard, the initial incubation was performed for 24 h, followed by removal of non-adherent cells, and the sessile cells were treated with LME^KAU0021^ (6496 to 6.34 µg/mL) for 24 h at 35 °C with shaking at 100 rpm.

Post-treatment, the metabolic activity of sessile cells was estimated using the XTT assay. For this purpose, post-incubation non-adherent cells were removed and sessile cells were washed thrice with PBS, followed by adding 91 µL of XTT (1 mg/mL; Sigma-Aldrich, USA) dissolved in PBS along with 9 µL of menadione (0.4 mM; Sigma-Aldrich USA) prepared in acetone and plates were then incubated for 2 h at 37 °C. The absorbance was recorded at 490 nm and the percentage biofilm inhibition was evaluated by using the below equation [33]. The lowest concentration of LME^KAU0021^ responsible for inhibition of ≥50% biofilm was considered as BIC_50_, whereas the concentration responsible for ≥90% inhibition was represented as BIC_90_.

% Biofilm inhibition=[(control OD490 nm−test OD490 nm)/control OD490 nm] × 100

To further understand and quantify the effect of LME^KAU0021^ on biofilm formation by *C. albicans* and *S. aureus*, individually and in combination, the crystal violet method was employed using a 96-well flat-bottomed microtiter plate. After growing the biofilms, plates were gently washed with methanol and stained with 0.1% crystal violet for 15 min. Following incubation, wells were washed with PBS and air-dried. In all the wells, 33% glacial acetic acid was added followed by reading the plates at 590 nm using a spectrophotometer (SpectraMax iD3 multi-mode microplate reader, Molecular Devices, San Jose, CA, USA).

### 2.7. Assessment of Microbial Cell Viability

The procedure used to evaluate the fungal and bacterial viability was adopted from Fernandes and co-workers [27]. As described previously, CFU was calculated to assess the cell viability in the biofilms formed by *C. albicans* and *S. aureus* individually and in combination. Post-adhesion, the cells were treated with sub-MIC concentrations of LME^KAU0021^ in respective growth medium and incubated for 24 h at 35 °C. Later on, the attached biofilm was washed twice with PBS, scraped out, and plated on SDA plates supplemented with 50 µg/mL chloramphenicol (Sigma-Aldrich, USA) for *C. albicans* and BHI agar plates supplemented with amphotericin B (Sigma-Aldrich, USA) for *S. aureus* for determining the mean log CFU/mL values [27].

### 2.8. Confocal Laser Scanning Microscopy (CLSM) of Mono- and Polymicrobial Biofilms

CLSM was used to validate the anti-biofilm property of LME^KAU0021^ on mature biofilms formed by mono- and polymicrobial settings, as described previously [34]. Briefly, mono- and polymicrobial biofilms were allowed to form on glass coverslips in 24-well microtiter plates under biofilm-forming conditions. The planktonic cells were removed after 24 h of incubation, followed by gentle washing of sessile cells with PBS. After that, LME^KAU0021^ at various concentrations (BIC_50_ and BIC_90_) was added to the designated wells except control wells (untreated cells) and the plates were incubated for 24 h at 37 °C. The planktonic cells were then aspirated, and biofilms were gently washed twice with PBS (pH ~7.4). For staining, the coverslips were transferred to a new 6-well microtiter plate and incubated with 2 mL PBS containing fluorescent dye FUN-1 (10 µM; Invitrogen, Thermo Fisher Scientific, Waltham, MA, USA, ZA; excitation wavelength = 543 nm and emission wavelength = 560 nm) and concanavalin A (ConA)-Alexa Fluor 488 conjugate (25 µg/mL; Invitrogen, Thermo Fisher Scientific, ZA; excitation wavelength = 488 nm and emission wavelength = 505 nm) and plates were incubated for 45 min at 37 °C in dark. After incubation, the glass coverslips were observed using a Zeiss Laser Scanning Confocal Microscope (LSM) 780 and Airyscan (Carl Zeiss, Inc., Jena, Germany). The confocal images of red (FUN-1) and green (ConA) fluorescence were recorded simultaneously using a multitrack mode.

### 2.9. Scanning Electron Microscopy (SEM) of Mono- and Polymicrobial Biofilms

SEM was performed to assess further the biofilm inhibition potential of LME^KAU0021^ against *C. albicans* and *S. aureus* single and mixed biofilms. Both the test pathogens were grown under biofilm formation conditions either in the presence of LME^KAU0021^ (BIC_90_) or in its absence (negative control), as described in the previous section. The adhered cells were washed with PBS, fixed with glutaraldehyde (5%), and subjected to gradient dehydration using ethanol (20, 40, 60, 80, and 100%). The samples were then immersed in hexamethyldisilazane (HMDS) and dried overnight at room temperature. The glass coverslips containing biofilms were mounted on carbon-coated aluminium stubs, and observed under SEM (Zeiss Gemini 2 Crossbeam 540 FEG SEM).

### 2.10. Assessment of Microbial Cell Membrane Integrity

For insight into antimicrobial mechanisms, the cellular membrane integrity of *C. albicans* and *S. aureus* (single and mixed culture conditions) was evaluated by propidium iodide (PI, Sigma-Aldrich, USA) staining method by following the protocol described elsewhere [35]. Briefly, both bacterial and yeast cells, individually and mixed, were inoculated in respective media and incubated at 37 °C, 150 rpm for 24 h. The cells were then spun, and pellets were washed with PBS, followed by adding fresh SDB and exposed to the LME^KAU0021^ (MIC) at standard conditions for 4 h. For this purpose, H_2_O_2_ (30%) and unexposed cells were used as positive and negative controls, respectively. Following exposure, cells were given PBS washing, stained with PI (30 μM) and kept at room temperature for 30 min away from light. Post-staining, cells were collected, washed with PBS and mounted on the slides, and viewed under fluorescence microscopy (Zeiss Laser Scanning Confocal Microscope (LSM) 780 and Airyscan (Carl Zeiss, Inc.).

### 2.11. Cytotoxicity Studies

The hemolytic assay was performed on horse red blood cells (RBC; NHLS, Johannesburg, South Africa) to evaluate the cytotoxic effect of the LME^KAU0021^ using a previously reported method [36]. Briefly, RBC suspension was prepared in PBS, and was treated with different concentrations of LME^KAU0021^ (0.25 × MIC, 0.5 × MIC, MIC and 2 × MIC) for 4 h at 37 °C. After incubation, samples were centrifuged (2000 rpm for 10 min). The supernatant was transferred to a 96-well microtiter plate to measure the absorbance spectrophotometrically (450 nm; SpectraMax iD3 multi-mode microplate reader, Molecular Devices, CA, USA). Triton X-100 (1%) and PBS were used as positive and negative controls, respectively, and the following equation was applied to calculate the percentage of hemolysis:

$$\%\;haemolysis=\left[\frac{A450\;of\;test\;compound\;treated\;sample-A450\;of\;buffer\;treated\;sample}{A450\;of\;1\;\%Triton\;X-100\;treated\;sample-A450\;of\;buffer\;treated\;sample}\right]\times 100\%$$

### 2.12. GC-MS Analysis

The GC-MS analysis was carried out by using the same protocol and instruments as depicted in our previously published work [37].

### 2.13. Statistical Analysis

Each experiment was conducted two times in triplicate and data analysed using one-way ANOVA test by Graph Pad Prism version 9.1.0 and the *p*-value ≤ 0.01 was considered as statistically significant.

## 3. Results and Discussion

*Lactiplantibacillus plantarum* KAU007 was previously isolated from camel milk with antiviral properties against the influenza virus H1N1 [23]. KAU0010 and KAU0021 isolated from fermented dates were identified as *Lactiplantibacillus pentosus* and *Limosilactobacillus fermentum*, respectively. The 16S rRNA gene sequences of KAU0010 and KAU0021 were submitted in GenBank with OP484948 and OP484972 accession numbers, respectively. *Lactobacillus sakie* Probio-65 is a Kimchi isolate, and a well-known probiotic strain which was used as a reference strain [38,39,40,41,42].

### 3.1. Lactobacillus Metabolite Extracts and Their Antimicrobial Activity against Planktonic Cells

The LMEs from different stains of *Lactobacillus* were extracted in organic solvents of varying polarity, as discussed elsewhere [43]. Different metabolites have been obtained from different LAB isolates, which are strain-specific and depend on the conditions under which the metabolites are extracted. Specific procedures involving precipitation, extraction, and isolation of antimicrobial metabolites such as bacteriocins or other AMPs from LAB have also been reported. In this study, we chose organic solvents of varying polarity to extract the majority of the metabolites, and found that the ethylacetate fraction contained the majority of the metabolites, which were long chain saturated and unsaturated fatty acids, hydroxy acids, carboxylic and dicarboxylic acid derivatives, aldehydes and amino acid derivatives, as confirmed by GC-MS. The final yield of the LMEs from the cell-free supernatant, obtained in the ethyl acetate fraction, was 0.22 g/mL, 0.16 g/mL, 0.11 g/mL, and 0.05 g/mL for LME^KAU0021^, LME ^KAU0010^, LME^KAU007^, and LME^Pro−65^, respectively.

All the LMEs were found to be active against planktonic cells of yeast and bacteria, both in single and mixed culture environments. The MIC and MFC values of test LMEs are compiled in Table 1, whereas the MIC values of fluconazole and azithromycin against *C. albicans* SC5314 and *S. aureus* ATCC29213 were recorded as 0.5 μg/mL. On the other hand, high MIC values were recorded under mixed culture conditions for both fluconazole and azithromycin, which were calculated as 4 μg/mL and 1 μg/mL, respectively.

The LMEs were found to be active against the planktonic cells of both pathogens in mono- and polymicrobial conditions; however, the LME^KAU0021^ was found to have stronger antimicrobial activity with the lowest MIC values against both the tested pathogens. Whereas the planktonic cells under mixed conditions were found less susceptible to the tested antimicrobials, the present finding is in agreement with the previous study [44]. The antifungal and antibacterial properties of *Lactobacillus* strains have been widely reported in the literature, suggesting their potential in antimicrobial drug discovery [45,46,47,48]; however, none of the findings discuss the anti-biofilm activity of these strains or their metabolites against mono- and polymicrobial biofilms formed by *C. albicans* SC5314 and MRSA.

The most frequently isolated fungal and bacterial pathogens from bloodstream infections are *C. albicans* and *Staphylococcus* species. Over 20% of bloodstream infections caused by *C. albicans* are polymicrobial, with *S. epidermidis* and *S. aureus* being the first and the third most repeatedly co-isolated commensal microbes, especially in immunocompromised patients [5]. These pathogenic microbes are known for their propensity to form biofilms inside the host and on abiotic medical devices. Therefore, the interaction between these microbes may raise virulence and drug resistance. Hence, inhibiting these mixed biofilms is the most important factor in developing an appropriate treatment strategy.

### 3.2. Effect of LME^KAU0021^ on Biofilms

The anti-biofilm activity of LME^KAU0021^ was quantified in terms of the reduced metabolic activity of mono- and polymicrobial biofilms compared to control using an XTT reduction assay. As reflected in Table 2, LME^KAU0021^ at various sub-inhibitory concentrations significantly reduced the *C. albicans* and *S. aureus* biofilms, both in the formation and 24 h preformed as compared to the control. The effect of LME^KAU0021^ on the inhibition of biofilm formation and preformed biofilms varies between bacteria and yeast, with the latter having one-fold higher biofilm inhibitory concentrations (BIC). At a concentration of 203 µg/mL of LME^KAU0021^, inhibition in biofilm formation of mixed culture was more than 91 ± 0.16%. Additionally, at 406 µg/mL, the inhibition of 24 h preformed biofilm of mixed culture was more than 94 ± 0.66% suggesting a relevant reduction compared to the control.

**Figure 1:**
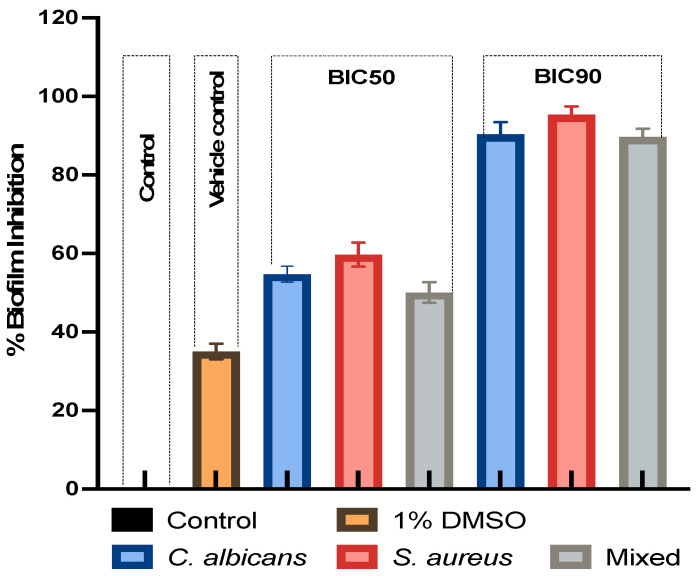
Percent inhibition of biofilm formation in *C. auris* and *S. aureus* alone and mixed species. Violet crystal (VC) staining method was adopted to evaluate the biofilm LME^KAU0021^ at various concentrations (BIC50 and BIC90). Percent inhibition was calcluated with respect to untreated control which was considered 0% inhibtion.

The effect of the LME^KAU0021^ on biofilm formation was determined by crystal violet staining and it was observed that total biomass formation in both individual and combined culture biofilms was lowered significantly (Figure 1). The capability of biofilm formation by *C. albicans* and *S. aureus* was altered after being treated with LME^KAU0021^ at different biofilm inhibitory concentrations. The results demonstrated a marked inhibition in biofilm formation that was higher in *S. aureus* than *C. albicans*. In mixed biofilm, the effect was slightly lower than for individual biofilms; however, significant inhibition was observed in both forms. The anti-biofilm property of LME^KAU0021^ against single and dual species further enhances its potential to be a perfect anti-biofilm agent because it will minimise the chance of developing resistance in these species under polymicrobial conditions.

*C. albicans* and *S. aureus* displayed a strong ability to form biofilms individually and in mixed conditions. Previous studies have shown similar interactions between *Candida* and *Staphylococcus* [1,49,50]. Therefore, this study used these pathogens to understand the in vitro potency of LME^KAU0021^ to inhibit the yeast and bacterial cells in single and mixed conditions. The collaboration of *C. albicans* and *S. aureus* in biofilm formation are known to be synergistic [1]. Yeast helps bacteria enhance their growth and upregulate various pathogenic attributes, including drug resistance [51,52,53], and at the same time, *S. aureus* stimulates growth and morphogenesis in yeast [54,55]. Notably, the polymicrobial biofilms show an additional level of pathogenicity and antimicrobial resistance towards standard drugs compared to the respective monomicrobial biofilms [1] and their planktonic counterparts [56,57]. Targeting the interkingdom cellular interaction and disrupting the biofilms can be a good strategy to prevent the problems caused by mixed biofilms. Present results suggest that LME^KAU0021^, at its MIC values (406 μg/mL), is effective against both planktonic and sessile cells, and can tackle single and polymicrobial conditions. This characteristic gives these LMEs an additional advantage over commonly used antimicrobials which have a narrow spectrum of antimicrobial properties and are, therefore, often associated with drug resistance.

### 3.3. Assessment of Cell Viability of C. albicans and S. aureus in Single and Mixed Biofilms

**Figure 2:**
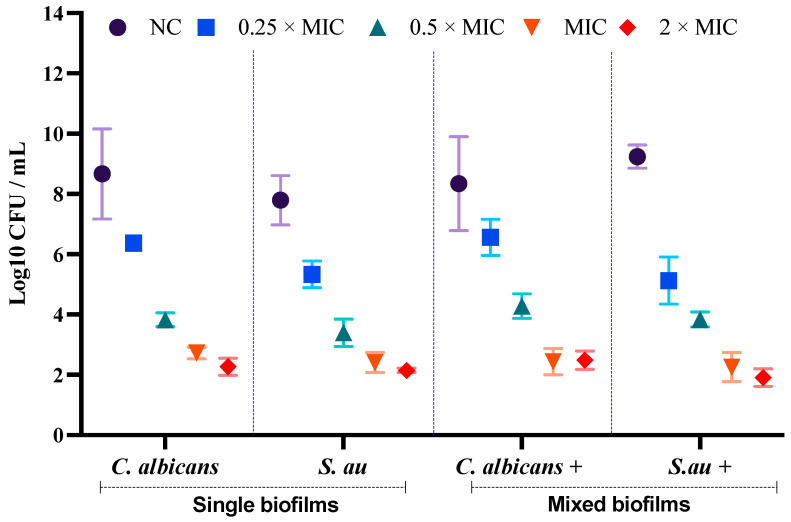
Viability assay of single- and mixed-microbial biofilms at different concentrations of the LME^KAU0021^. The polymicrobial biofilm cells of *C. albicans* and *S. aureus* (*C. albicans*+, viable cells of *C. albicans* in mixed biofilms; *S. aureus*+, viable cells of *S. aureus* in mixed biofilms). Negative control (NC), untreated single and mixed biofilm.

Besides metabolic activity, viability count also exhibited a concentration-dependent inhibition of formation and preformed biofilm in mono- and polymicrobial by LME^KAU0021^ as the CFU count was significantly reduced in both types of biofilms with the increasing concentration of LME^KAU0021^ (Figure 2). In the presence of 408 µg/mL of LME^KAU0021^, the log_10_ CFU value for *C. albicans* cells in a single biofilm was reduced from 8.66 (negative control) to 2.72 (2 × MIC). These results for *S. aureus* in the presence of 101.5 µg/mL were lowered from 7.79 (negative control) to 2.40 (2 × MIC). At 408 µg/mL (MIC), LME^KAU0021^ showed a potential reduction in the log_10_ CFU value of *C. albicans* and *S. aureus* in the polymicrobial biofilms as displayed by the reduction from 8.34 to 2.44 and from 9.23 to 2.25 for *C. albicans* and *S. aureus*, respectively. Furthermore, to understand the mode of antimicrobial action of LME^KAU0021^ on the biofilm structure, CLSM and SEM analysis was performed.

### 3.4. Confocal Laser Scanning Microscopy (CLSM) of Mono- and Polymicrobial Biofilms

**Figure 3:**
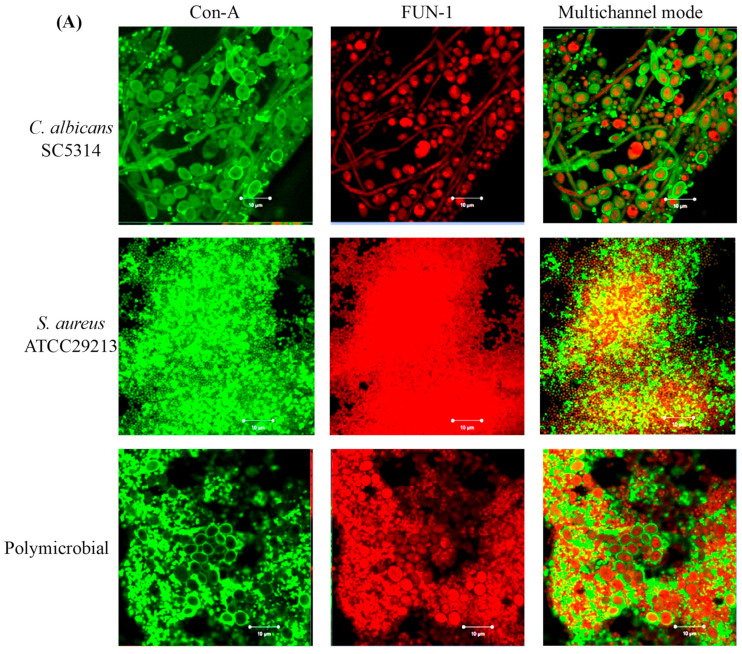

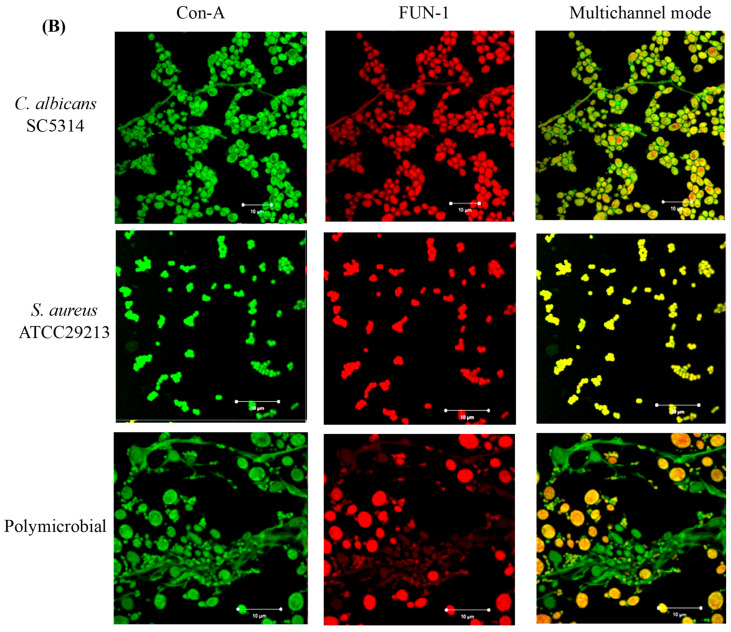

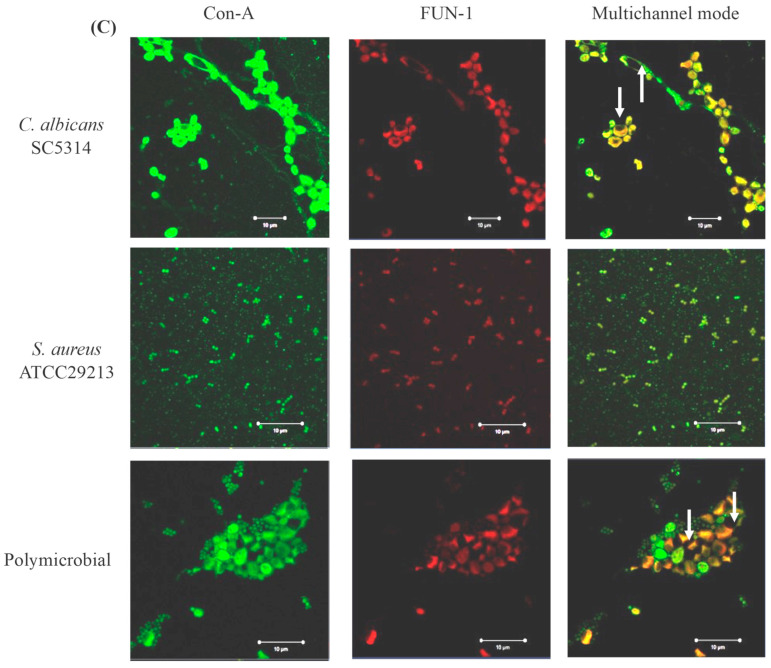
CLSM analysis. The figure represents the anti-biofilm potential of LME^KAU0021^ against 24 h mature mono- and polymicrobial biofilms formed by *C. albicans* SC5314 and *S. aureus*. The image was taken at 63× oil immersion objective with magnification, ×3.0. The structures in green represent the biofilm matrix; yellow-green are metabolically inactive cells; orange-red are the viable cells. (**A**) untreated control; (**B**) biofilm exposed to BIC_50_ of LME^KAU0021^; (**C**) biofilm exposed to BIC_90_ of LME^KAU0021^; white arrow shows the groove and damage within the cell.

For CLSM, a combination of fluorescent stains FUN-1 (cytoplasmic probe for cell viability) and Con-A (specifically associated with the cell wall polysaccharides) was utilized. The presence of solid green fluorescence depicted the binding of Con-A to the cell wall polysaccharides in mono- and polymicrobial biofilms, whereas FUN-1 stained the metabolically active cells. Therefore, areas fluorescing orange-red represent the presence of metabolically active microbial cells. On the other hand, the biofilm matrix is represented by green fluorescence, whereas, in dead cells, FUN-1 remains in the cytosol and fluoresces yellow-green [58]. The images obtained for untreated control (Figure 3A) clearly showed dense and compact biofilm architecture, which can be seen as red-green fluorescence in the multichannel mode. Conversely, biofilms exposed to BIC_50_ (Figure 3B) and BIC_90_ (Figure 3C) of LME^KAU0021^ showed a reduction in biofilm density and in live cells. Thus more yellow-green fluorescence representing non-viable cells was observed (Figure 3B,C). The damage in the biofilm was found to be concentration-dependent, as at BIC_90,_ the cell size was reduced, depressions were clearly seen, and faint red fluorescence advocated the non-viability of cells embedded in mono- and polymicrobial biofilms.

### 3.5. SEM Analysis of Mono- and Polymicrobial Biofilms

**Figure 4:**
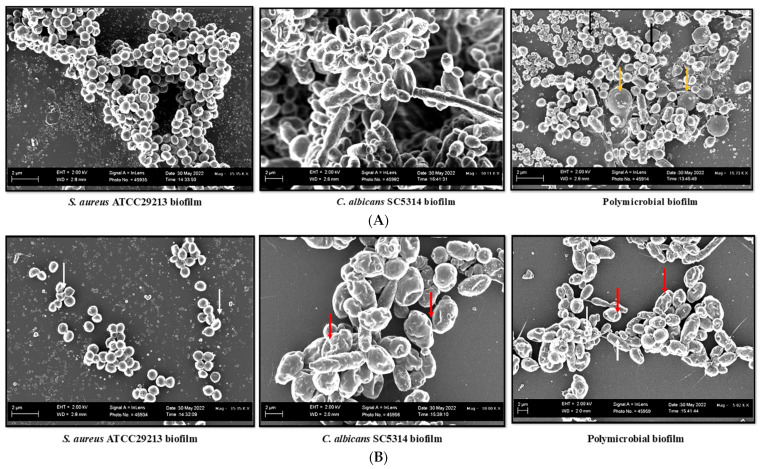
SEM images of mono- and polymicrobial biofilms formed by *C. albicans* and *S. aureus*. (**A**) untreated; (**B**) treated with LME^KAU0021^ at BIC_90_. Black and yellow arrows show the presence of *S. aureus* and *C. albicans*, respectively, in the untreated control mixed biofilm. White and red arrows point out the deformity in the *S. aureus* and *C. albicans* respectively in the LME^KAU0021^ treated mixed biofilms.

A complex three-dimensional network of cells was observed in the untreated controls both in mono- and polymicrobial biofilms (Figure 4A). In contrast, cell distortion, shrinkage, and the presence of grooves were observed in single biofilms formed by *C. albicans* (red arrow) and *S. aureus* (white arrow) at BIC_90_ of LME^KAU0021^, while the abrogation of polymicrobial biofilms was also observed at the same concentration of LME^KAU0021^. Furthermore, the sessile cells present in the biofilm were distorted from thier original shape (Figure 4B).

SEM analysis helped us understand the effect of LME^KAU0021^ on the morphology of cells embedded in mono- and polymicrobial biofilms. The results showed the difference between treated and untreated biofilms in single and mixed biofilm conditions. The biofilm formed by *C. albicans* was dense and full of hyphae and yeast cells and accompanied by a good amount of matrix, and the results are in agreement with previous findings [59]. The biofilm formed by *S. aureus* also had a dense cellular architecture with compact cells, which is in further accord with similar findings by Guo and co-workers [60]. In addition, a synergistic interaction between *C. albicans* and *S. aureus* was observed in the polymicrobial biofilms where *S. aureus* cells were entangled between and around *C. albicans*. A recent study by Scheunemann and co-workers [56] also studied the positive interaction of these microbes in mixed biofilms. In the untreated control mixed biofilms, significant amounts of extracellular matrices were found, which could be related to the increased antimicrobial resistance in mixed culture conditions. Similar observations were also observed in a recent study where enhanced biofilm matrix production observed in mixed biofilms was the reason for amplified drug resistance [61]. On the other hand, treatment with LME^KAU0021^ caused damage to the cell shape. In addition, it reduced the biofilm matrix that allowed the penetration of LME^KAU0021^ into the cells embedded in the biofilm, resulting in the abrogation of the mixed biofilm.

### 3.6. Assessment of Microbial Cell Membrane Integrity

**Figure 5:**
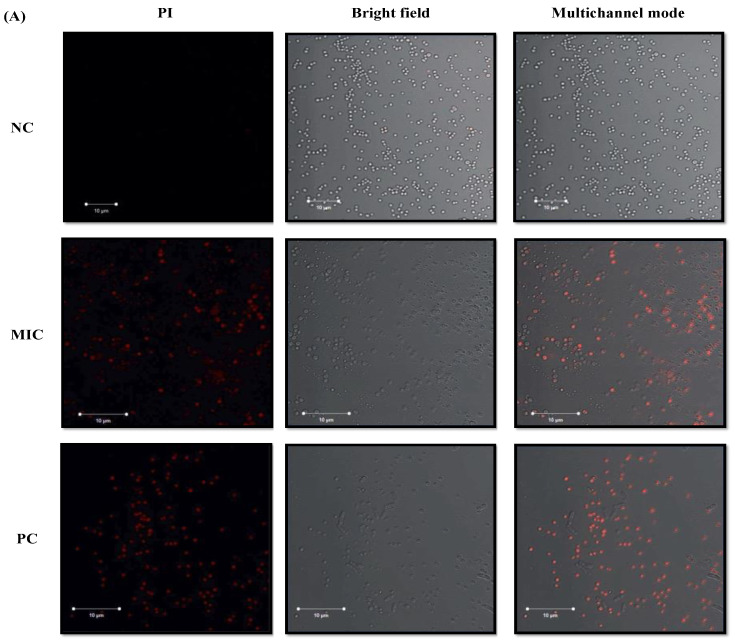

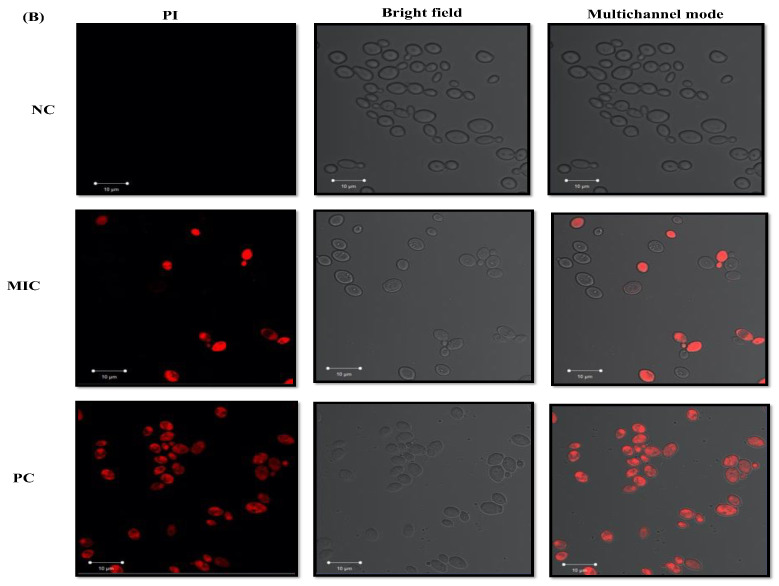

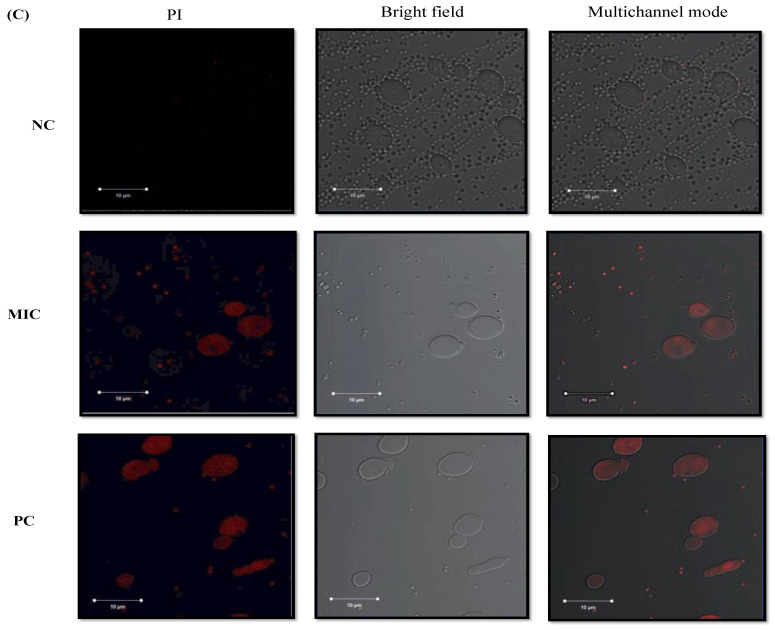
Cell membrane integrity monitored PI. Exposure of *C. albicans* and *S. aureus* in single and mixed culture condition by LME^KAU0021^ (MIC). (**A**) *S. aureus* cells (**B**) *C. albicans* (**C**) mixed culture conditions. NC, negative control; PC, treatment with H_2_O_2_. Cells were observed by fluorescence microscopy (63× oil immersion objective).

Plasma membranes protect microbes from external stress conditions; therefore, compounds targeting the plasma membrane can be used as potential candidates for antimicrobial therapy. In this study, PI is used to track the membrane-disruption ability of LME^KAU0021^, and cells with red fluorescence were recorded as PI-positive, indicating defects in the cell membrane [62]. Exposure to LME^KAU0021^ at the MIC value resulted in compromised cellular membranes, both in single and mixed culture conditions, which was shown by a higher number of PI-positive cells and was similar to positive control and contrary to untreated control (Figure 5). The cell envelopes of *C. albicans*, and *S. aureus* in negative control were found intact, and therefore no fluorescence was recorded. Similarly, both pathogens were healthy under mixed culture conditions, and consequently, no PI-positive cells were observed during microscopy. However, treatment with H_2_O_2_ (positive control) resulted in a compromised cell wall/membrane, and thus cellular uptake of PI was recorded in terms of PI-positive cells. Therefore, the antibacterial and antifungal properties of LME^KAU0021^ can be advocated by its ability to rupture the cell envelope, interact with intracellular components and trigger cell content leakage. These results also support the CLSM and SEM observation and highlight the probable mode of action of LME^KAU0021^ by causing a change in cell numbers, distorting cell morphology, and lowering biofilm matrix production in mono- and polymicrobial biofilms.

### 3.7. Cytotoxicity

**Figure 6:**
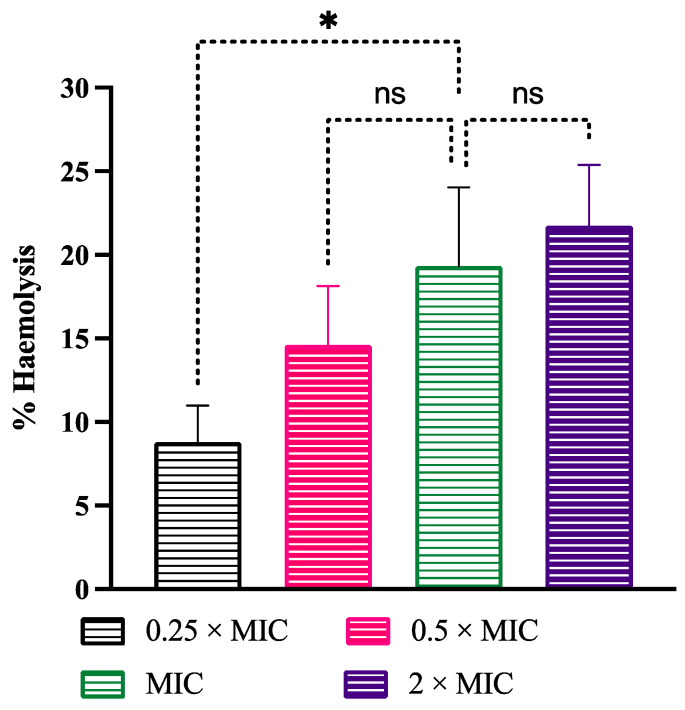
Hemolytic activity. Hemolysis of RBCs by LME^KAU0021^ at various concentrations. 1% Triton X-100 (Positive control, 100% hemolysis); PBS, negative control. * *p*-value ≤ 0.0224. ns = not significant.

Hemolysis is one of the well-known attributes used to check the safety of drugs. To check the safe use of LME^KAU0021^, hemolysis was evaluated using RBC. For comparison, PBS and Triton X-100 were used as negative and positive controls, causing 0% and 100% hemolysis in RBC, respectively. The LME^KAU0021^ at various concentrations showed hemolysis ranging from 8.79% to 21.74% (Figure 6). Our results agree with previous findings where no evidence of hemolysis was observed from probiotic *Lactobacillus* strains [46]. However, some strains have been reported to show α-hemolysis [63]. Other researchers also supported the fact that food grade *Lactobacillus* strains rarely display hemolytic activity [64,65]. All these findings advocated the low cytotoxicity of LME^KAU0021^ and suggest its use for in vivo experiments.

### 3.8. GC-MS

**Figure 7:**
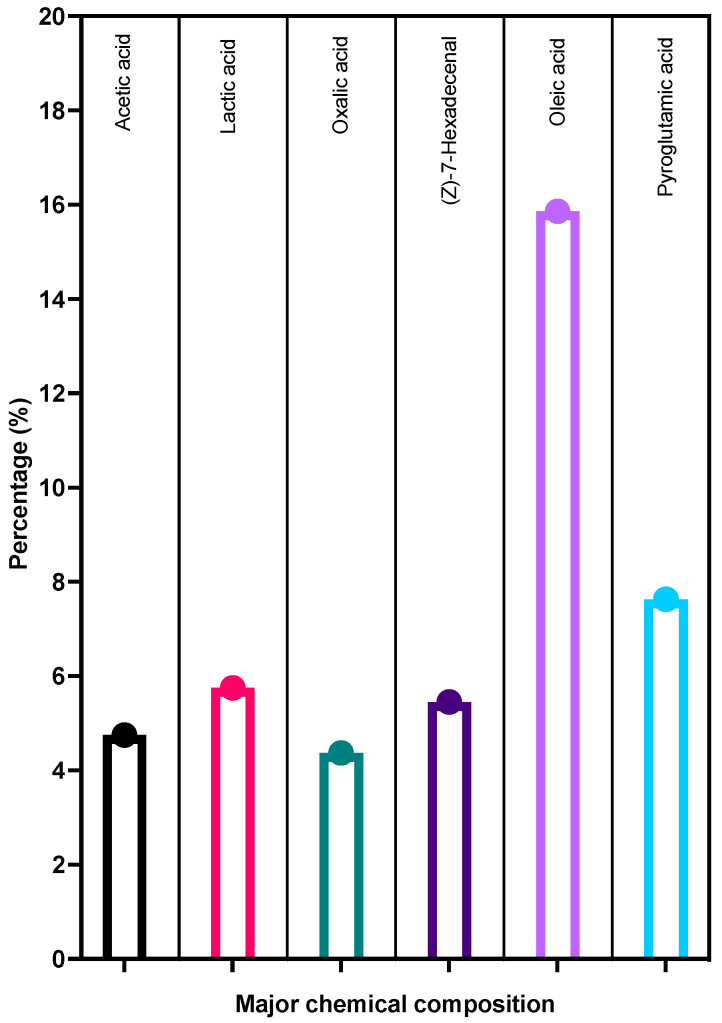
The GC-MS chemical composition of LME^KAU0021^ dervied from *Limosilactobacillus fermentum* KAU0021. The bars depict compounds of major composition found in LME^KAU0021^.

Based on the GC-MS analysis of LME^KAU0021^, thirty-two compounds were identified, accounting for 88.98% of the total LME ^KAU0021^. As shown in Figure 7, six compounds were identified as the major composition of the extract. Figure S1 shows the metabolic profile obtained from a typical GC-MS total ion chromatogram (TIC). Table S1 illustrates the composition of LME ^KAU0021^, which includes long-chain saturated and unsaturated fatty acids, carboxylic and dicarboxylic acids, hydroxy acids, amino acids, and various compounds in small amounts.

## 4. Conclusions

The present study reports the antimicrobial potency of four *Lactobacillus* species. Out of four LMEs, LME^KAU0021^ displayed strong antimicrobial activity against planktonic cells of *C. albicans* and *S. aureus* in single and mixed culture conditions. As a result, the extract significantly reduces microbial viability within monomicrobial and polymicrobial biofilms formed by *C. albicans* and *S. aureus*. The presence of bioactive compounds in LME^KAU0021^ has not been fully characterized; however, this study represents one of a few to describe its anti-biofilm potency against mono- and polymicrobial biofilms formed by *C. albicans* and *S. aureus*. The present findings open a window for more detailed studies to discover an alternative strategy to combat serious polymicrobial infections.

## Figures and Tables

**Table 1:** The MIC and MFC/MBC values of test LMEs against *C. albicans* SC5314 and *S. aureus* ATCC29213.

Pathogens	LME^KAU0021^ (μg/mL)		LME^KAU0010^ (μg/mL)		LME^KAU007^ (μg/mL)		LME^Pro−65^ (μg/mL)	
	MIC	MFC/MBC	MIC	MFC/MBC	MIC	MFC/MBC	MIC	MFC/MBC
*C. albicans*	406	812	406	812	1624	3248	1624	>3248
*S. aureus*	203	406	406	812	1624	3248	1624	>3248
Polymicrobial	406	812	406	1624	3248	>3248	1624	>3248

**Table 2:** Biofilm inhibitory concentrations (BIC) of LME^KAU0021^ against single and mixed biofilms (during their formation and 24 h preformed) of *C. albicans* and MRSA.

LME^KAU0021^	Biofilm Formation			24 h Preformed Biofilm		
	*C. albicans* Alone	*S. aureus* Alone	Mixed Biofilms	*C. albicans* Alone	*S. aureus* Alone	Mixed Biofilms
BIC_50_	101.5 μg/mL	50.75 μg/mL	101.5 μg/mL	203 μg/mL	101.5 μg/mL	203 μg/mL
BIC_90_	203 μg/mL	101.5 μg/mL	203 μg/mL	406 μg/mL	203 μg/mL	406 μg/mL

## Data Availability

Not applicable.

## Supplementary Materials

The following supporting information can be downloaded at: https://www.mdpi.com/article/10.3390/pharmaceutics15041079/s1, Figure S1: Total ion chromatogram (TIC) of LME^KAU0021^. Table S1: Chemical composition of the LME^KAU0021^ obtained from *Limosilactobacillus fermentum* KAU0021.
