# Supplementary material for: Limosilactobacillus fermentum KAU0021 Abrogates Mono- and Polymicrobial Biofilms Formed by Candida albicans and Staphylococcus aureus
Source: Pharmaceutics. 2023 Mar 27;15(4):1079. doi: 10.3390/pharmaceutics15041079 (PMC10145238; doi:10.3390/pharmaceutics15041079)
Supplement: Supplementary file 1 [file pharmaceutics-15-01079-s001.zip › pharmaceutics-2108891-SI.pdf]

Supplementary Data :

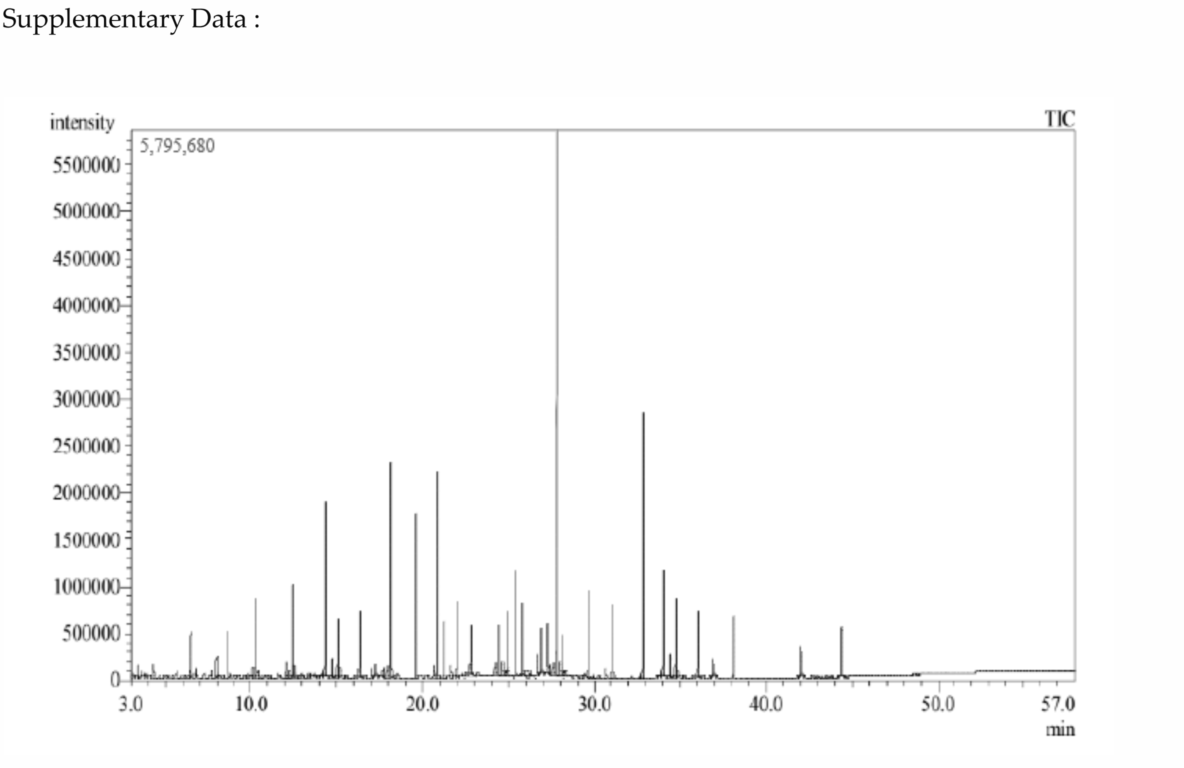

Figure S1: Total ion chromatogram (TIC) of LME^{KAU0021}.

Supplementary Data :

Table S1: Chemical composition of the LME^{KAU0021} obtained from *Limosilactobacillus fermentum* KAU0021

S.No.	Metabolite[#]	M.Wt	Retention time	Percentage
1	Octadecanoic acid	284.48	6.323	1.65±0.016
2	Hexadecanoic acid	256.43	7.957	0.85±0.041
3	Propanoic acid	118.13	8.801	1.64±0.016
4	Stearic acid	284.48	10.254	2.45±0.028
5	Myristic acid	228.37	12.634	2.85±0.008
6	**Acetic acid**	**132.15**	**14.254**	**4.75±0.018**
7	Phenylacetic acid	136.14	15.193	1.85±0.084
8	3-phenyllactic acid	166.17	16.327	1.94±0.048
9	**Lactic acid**	**90.08**	**18.133**	**5.75±0.020**
10	**Oxalic acid**	**90.03**	**19.756**	**4.37±.033**
11	**(Z)-7-Hexadecenal**	**238.41**	**20.931**	**5.45±0.021**
12	Phenol,2,4-bis(1,1-dimethylethyl)	278.5	21.131	1.55±0.028
13	4-aminobutyric acid	103.12	22.033	1.61±0.016
14	Sebacic acid	202.25	22.966	1.58±.032
15	3-hydroxybutyric acid	104.10	24.531	1.45±0.040
16	Hexadecanoic acid methyl ester	270.45	24.902	1.68±0.040
17	2-butenedioic acid	116.07	25.476	2.88±0.097
18	Phosphoric acid	97.99	25.875	1.96±0.048
19	2-hydroxyisovaleric acid	74.08	27.069	0.89±0.048
20	2-hydroxy isocaproic acid	122.12	27.243	0.94±0.032
21	**Oleic acid**	**282.47**	**27.911**	**15.86±0.013**
22	Valine	117.15	29.842	2.34±0.032
23	Malic acid	134.08	31.108	2.23±0.036
24	**Pyroglutamic acid**	**129.04**	**32.916**	**7.63±0.040**
25	Succinic acid	118.09	34.113	2.85±0.032
26	2,5-dihydroxyacetopheneone	152.15	34.523	0.87±0.040
27	Azelaic acid	188.22	34.921	1.97±0.043
28	Glutamic acid	147.13	36.116	1.84±0.016
29	Arabitol	152.14	37.052	0.88±0.040
30	Erythrose	120.1	38.125	1.68±0.008
31	Glucose	180.15	42.083	0.86±0.297
32	Citric acid	192.12	44.304	1.88±0.064
	**Total percentage identified**			88.98

[#]The metabolites are listed according to the elution order. Unknown compounds and metabolites in <0.80% concentration have not been listed.
